# Placental insufficiency irrespective of offspring karyotype in maternal Turner syndrome: a case series and literature review

**DOI:** 10.20945/2359-4292-2024-0144

**Published:** 2025-03-11

**Authors:** Beáta Vida, Gábor Méhes, Olga Török, Mónika Orosz, Zoárd Tibor Krasznai, Attila Jakab, Zsuzsanna Buczkó, Tamás Deli, Péter Juhász

**Affiliations:** 1 Department of Obstetrics and Gynaecology, University of Debrecen, Debrecen, Hungary; 2 Department of Pathology, University of Debrecen, Debrecen, Hungary

## Abstract

Turner syndrome is one of the most common aneuploidies. In vitro fertilization with oocyte donation is the usual method of assisted conception, but spontaneous pregnancy can also occur. Although pregnancies in Turner syndrome are widely accepted to be associated with small for gestational age foetuses, neither the causal role of placental insufficiency nor the contribution of maternal and foetal factors is well understood. Between 2009 and 2023, we followed 75 patients diagnosed with Turner syndrome at our university clinic, and four Turner syndrome patients became pregnant (4/75; 5.3%): ten pregnancies with seven live births (7/10; 70%) were reported. Conception was spontaneous in 6/7 patients (86%), and one patient had in vitro fertilization with oocyte donation. Two Turner syndrome patients with karyotype 45,X and two Turner syndrome patients with mosaicism (45,X/46,XX) were identified. Prenatal transabdominal amniocentesis revealed aneuploidy (45,X) in two foetuses. The most common obstetric complication was placental insufficiency, which presented as intrauterine growth restriction and foetal distress. Four early-term deliveries, one late-term delivery, one preterm delivery, and one extremely premature delivery occurred, and all pregnancies were terminated by caesarean section. No severe maternal complications during pregnancy were reported. Only newborns with Turner syndrome had long-term health problems. In Turner syndrome patients, even if pregnancy is conceived spontaneously, no maternal complications occur, and the foetus also has a normal karyotype, there is still a high prevalence of placental insufficiency and foetal compromise. The presented cases highlight the possible role of inherent maternal factors in Turner syndrome-associated intrauterine growth restriction and emphasize the importance of enhanced obstetric surveillance even in apparently uncomplicated Turner syndrome pregnancies.

## INTRODUCTION

Turner syndrome (TS) (^1^) is one of the most common aneuploidies, leading to a characteristic phenotype and a wide range of comorbidities. Its incidence is 1/2,500 live female births, which are generally caused by the complete or partial loss of one of the X chromosomes (45,X).

Although ovarian insufficiency and primary amenorrhea are among the leading symptoms of TS, approximately 5% of patients may become pregnant spontaneously (^2^-^4^). This may be due to mosaicism or the varied penetrance of the classical streek gonads. However, spontaneous pregnancy in TS cannot be facilitated effectively; thus, donor oocyte *in vitro* fertilization (IVF) has been the standard fertility treatment (^5^-^7^). According to the current guidelines (^8^,^9^), irrespective of the method of conception, multidisciplinary preconception counselling, including a cardiologic and a genetic counselling, as well as endocrinologic follow-up, is recommended for every TS patient considering pregnancy (^7^,^10^-^13^). Furthermore, enhanced and centralized obstetric care is also indicated since miscarriage, preeclampsia, preterm labour, stillbirth, and foetal growth restriction are also more common in TS pregnancies (^2^,^3^,^7^,^14^-^16^).

Despite the constantly growing knowledge regarding TS, the cause of intrauterine growth restriction in TS has not yet been elucidated, and the long-term outcomes of pregnancies in TS patients with respect to the karyotypes of the offspring are also lacking. Here, we present the cases of four TS patients and their medical history and course of pregnancy. To the best of our knowledge, this is the first case series of spontaneous pregnancies of TS patients in which maternal and foetal karyotypes, histology and fluorescence *in situ* hybridization (FISH) results from a maternal ovarian biopsy; evidence of placental insufficiency and placental histology; and shortand long-term outcomes of the newborns were reported at the same time.

## METHODS

### Patients and data collection

For our retrospective analysis, we collected data from our medical database of 75 patients treated for TS at the Department of Obstetrics and Gynaecology, University of Debrecen, between 1st January 2009 and 1st January 2023. All the pregnancies of TS patients in this period were included and analysed with patient consent. The outcomes of the ten pregnancies of the four enrolled patients were collected from hospital charts and the local (Med Solution, UDMed) and Hungarian national electronic medical databases (National eHealth Infrastructure; EESZT), including inand outpatient reports of gynaecologic, paediatric, neonatologic, endocrinologic, cardiologic, internal medicine and genetic consultations (ethical approval: DE RKEB/IKEB 5953-2022). Karyotyping of the pregnant patients was performed at diagnosis, when the first signs of primary ovarian insufficiency, short stature, or other phenotypic features were recognized. Preconceptionally, cardiologic counselling and cardiac imaging were performed, and routine paediatric endocrinologic, gynaecologic diagnostic workups and treatment protocols were followed. Newborns were categorized by gestational age: extremely preterm newborns were born before 28 weeks gestation, preterm infants were born between 28 weeks and 36 weeks and 6 days gestation, and early-term infants were born between 37 weeks and 38 weeks and 6 days gestation. During pregnancy, all patients underwent official routine prenatal care according to the national guidelines and received special multidisciplinary surveillance throughout pregnancy, including obstetric, genetic, endocrinologic and cardiologic examinations. Echocardiography was performed preconceptionally and during pregnancy, with the possibility of referral for transoesophageal echocardiography in cases of suspected aortic abnormalities. This, however, did not become necessary. In three of the four patients that spontaneous conception was registered, amniocentesis was performed in the second trimester for the foetal karyotype after genetic counselling.

### Karyotyping and cytogenetic testing

The samples were evaluated at the Cytogenetics Laboratory, Department of Obstetrics and Gynaecology. One patient underwent IVF at a foreign hospital in Europe, where the oocyte of the patient’s sister was fertilized and transferred.

Foetal cytogenetic analysis during pregnancy was performed using amnionic fluid samples, but chorionic villus sampling could be carried out. The culture setup for prenatal diagnosis was long-term cell culture to allow for mosaicism exclusion. Long-term cell cultures were used to determine the foetal karyotype, but they could also be used for further cytogenetic or molecular analysis. In some cases that an urgent result was needed, both chorionic villus and amnionic fluid samples were tested, first to achieve quick direct diagnosis and second to obtain cell cultures.

Maternal cytogenetic analysis was performed using peripheral lymphocytes from blood samples. The karyotypes of the mothers were determined before their pregnancies. In adult patients, at least 30 lymphocytes were used. In ambiguous cases or when mosaicism was found, FISH examination was also performed to confirm the result. Every karyotype analysis was carried out using a G-banding technique. For staining, Giemsa dye was used to improve the resolution of individual bands, and the samples were analysed via light microscopy at 1,000x magnification after protein digestion with trypsin.

### Histology

In one patient (Patient 1), the placenta and the umbilical cord were sent for routine histology. The karyotype of the patient determined by the technique described above was known before the surgery. During the caesarean section, a biopsy sample was taken from one of the maternal ovaries, and routine histologic evaluation with haematoxylin and eosin (H&E) staining and X chromosome centromere-specific FISH was performed on this sample.

Fluorescence in situ hybridization was performed with MetaSystems CEP X Orange Probe according to the local protocol of the Department of Pathology. The formalin-fixed, paraffin-embedded slides were dried before pretreatment with a mixture of FISH probes and then covered with a cover slip. After the slide was denatured at 75 °C for 2 minutes, it was incubated at 37°C overnight in a wet cabin, followed by posthybridization washing while maintaining the pH and temperature. Counterstaining was performed with 10 µl of 4′,6-diamidino-2-phenylindole (DAPI) for 10 minutes. The slides were stored at -20 °C in the dark until evaluation. The signals were counted via a fluorescence laser microscope equipped with appropriate excitation and emission filters with different wavelengths, allowing visualization of the blue nucleus and the red hybridization signal of the centromere region of chromosome X. Microscopic evaluation of FISH was performed on 50 ovarian cells (80% stromal cells and 20% follicular cells).

### Presentation of cases and results

We collected data from our database of 75 patients treated for TS at the Department of Obstetrics and Gynaecology, between 2009 and 2023. All the pregnancies of TS patients in this period were included and analysed.

Only 12/75 patients (16% of the cohort) experienced spontaneous menarche. Among our pregnant patients, all patients, except Patient 1, experienced spontaneous menarche. We identified ten pregnancies resulting in seven live births (7/10; 70%), two early miscarriages (2/10, 20%) and one artificial abortion (1/10; 10%) in four patients (4/75; 5.3%) **(Table 1)**. According to our data, 2/4 of the patients had TS with karyotype 45,X, whereas the other two patients had TS with mosaicism (45,X/46,XX). Maternal TS was diagnosed in early childhood in one patient, around the expected time of menarche in two patients, and in late adolescence in the fourth patient. All patients had short stature, and three of the patients had formerly received growth hormone treatment because of short stature. In examining the most common phenotypic alterations, in the case of Patient 1, we did not register any features related to TS except for short stature. However, in the other pregnant patients, minor TS-related stigmas, e.g., low-set ears, dysmorphic faces, pterygium colli, or hypertrichosis, were observed. Maternal comorbidities were mostly cardiovascular disorders, such as hypertension, mitral valve prolapse, and tachycardia, but severe structural heart anomalies were not present. Hypothyroidism, benign frontal haemangioma (followed by magnetic resonance imaging), and a benign breast tumour were also diagnosed, treated and followed-up preconceptionally.

**Table 1:** Patient characteristics and medical histories

Patient No.	Age at (last) delivery (years old)	Age at diagnosis (years old)	Karyotype	Menstrual cycles	Adult height (cm)	Adult BMI (kg/m^2^)	Dysmorphological features	Concomitant diseases and treatments	Obstetric history
1	20	1	45,X/46,XX^*^	Irregular	149	24.3	Short stature (GH therapy)	Benign frontal haemangioma Tachycardia	G:2, P:1^†^
2	34	17	45,X	Primary amenorrhoea	154	20.9	Short stature (GH therapy) Dysmorphic face, low-set ears, clitoromegaly	Mitral prolapse Hypothyroidism Benign breast tumour	G:1, P:1
3	25	12	45,X	Irregular	149	20.8	Short stature (GH therapy) Dysmorphic face, low-set ears, hypertrichosis, *pterygium colli, acanthosis*	Benign frontal haemangioma Tachycardia	G:2, P:2
4	25	12	45,X/46,XX	Irregular	150	22.7	Underdeveloped breasts, dysmorphic face, low-set ears	Mitral prolapse Hypertension Allergic asthma Otologic surgery	G:5, P:3^‡^

* Histology of ovary at caesarean section: stroma-rich ovarian tissue, 50% stroma, 50% regular ovarian tissue with some sporadic primordial follicles.

X chromosome centromere specific fluorescence *in situ* hybridization of somatic cells of ovarian tissue: 0 hybridization signal with 80%, 1 hybridization signal with 13.2%, 2 hybridization signals with 6.8%;

† Patient 1 had one artificial abortion;

‡ Patient 4 had two early miscarriages.

BMI: body mass index; GH: growth hormone; G: gravidity; P: parity.

Three of the four patients conceived spontaneously. In these cases, after genetic counselling, amniocentesis was performed in the second trimester for the foetal karyotype. The results are shown in **Table 2**. One patient underwent IVF with donated oocytes from the patient’s sister, and preimplantation genetic testing for aneuploidies (PGT-A) revealed a normal karyotype. During pregnancy, all patients underwent routine prenatal care according to the national guidelines (^17^), and they received special multidisciplinary surveillance throughout pregnancy, including obstetric, genetic, endocrinologic and cardiologic examinations (^8^).

**Table 2:** Obstetric outcomes of Turner syndrome pregnancies and neurodevelopmental outcomes and comorbidities of the offspring

Patient No.	Pregnancy No.	Mode of conception	Time of delivery (gestational week)	Obstetric complication	Mode of delivery	Birth weight	Karyotype of offspring	Neurodevelopment and comorbidities of offspring
1	1	Spontaneous	37	IUGR, oligohydramnios^*^	C/S	1,880 g	46,XY	Normal development
2	1	IVF (DO, sister)	39	Unstable lie	C/S	3,520 g	46,XY	Normal development
3	1	Spontaneous	38	IUGR, foetal distress	C/S	2,030 g	45,X	Short stature, Strabism
	2	Spontaneous	24	IUGR, oligohydramnios Foetal distress	C/S	300 g	45,X	Somatomental retardation, short stature, Hypothyroidism, Ophthalmological disorders
4	1	Spontaneous	36	IUGR, foetal distress	C/S	2,360 g	46,XX	Normal development
	2	Spontaneous	38	IUGR, foetal distress	C/S	2,720 g	46,XY	Normal development
	3	Spontaneous	38	IUGR, transverse position	C/S	2,070 g	46,XX	Normal development

* Histology of placenta and cord: negative.

IVF: *in vitro* fertilization; DO: donor oocyte, IUGR: intrauterine growth restriction; C/S: caesarean section.

**Figure 1:**
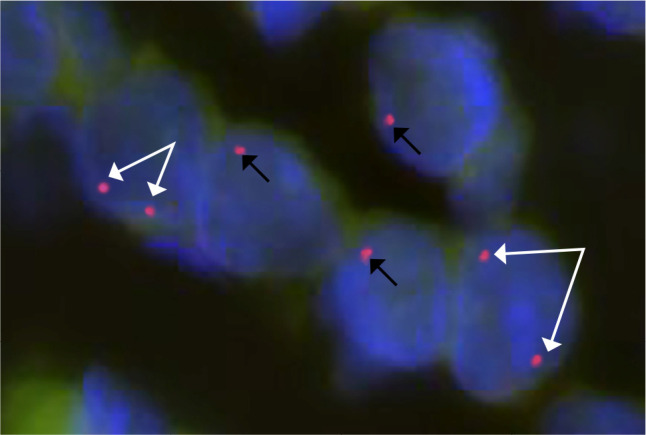
Fluorescent *in situ* hybridization of the ovarian biopsy specimen of Patient 1. Evaluation was performed with fluorescence *in situ* hybridization (chromosome X, centromere region red signal, nucleus blue signal). Picture represents five cells: two of five cells contain two (white arrows), and three of five cells contain one hybridization signal (black arrows).

Patient 1 had two pregnancies, one early-term delivery and one artificial abortion. Following the delivery of an IUGR foetus via a caesarean section, placental and cord histology was carried out, and during the caesarean section, an ovarian biopsy was also performed for histology and FISH. The former revealed normal placental and umbilical cord structures, whereas the maternal ovaries were very rich in stroma (50% stroma, 50% regular ovarian tissue), and some sporadic primordial follicles could also be identified. Fluorescence *in situ* hybridization confirmed ovarian mosaicism. **Figure 1** shows a representative image of the hybridization signals. Overall, 13.2% of the somatic (stromal) cells presented one hybridization signal, and 6.8% presented two hybridization signals with X chromosome centromere-specific FISH. Oocytes could not be analysed, as their numbers were so low that they were morphologically difficult to identify.

Patient 2 had the only normal-weight and late-term newborn who conceived after oocyte donation and IVF. The indication for caesarean delivery was not foetal distress but an unstable lie.

Patient 3 had an early-term and extremely preterm birth with an extremely low birth-weight newborn. In the latter case, the patient arrived at our clinic because of the lack of foetal movements in the 24th week of gestation. On examination, severe growth restriction and oligohydramnios were recognized. Steroid infant respiratory distress syndrome (IRDS) prophylaxis was initiated, but soon after admission to the delivery room, a category III foetal heart rate pattern was recorded on the cardiotocogram. Thus, an emergency caesarean section was performed, and a 300-g extremely premature baby was born. Following the routine steps of care for extremely low birth weight infants in the neonatal intensive care unit (NICU), the general condition of the baby improved, and several months after delivery, the infant was taken home.

Patient 4 had a total of five pregnancies with two early miscarriages, two early-term deliveries and one late preterm delivery. In the latter case, which was monitored for severe intrauterine growth restriction and oligohydramnios, an emergency caesarean section was performed due to severe foetal distress registered by cardiotocography.

Except for the first pregnancy of Patient 3, all pregnancies were observed at our high-risk pregnancy ward during the third trimester and at the delivery room. During the observation period, routine ultrasound, Doppler ultrasound, nonstress tests, and biophysical profile (BPP) analyses were carried out. In the case of the second pregnancy in Patient 3, an urgent caesarean section was performed soon after admission to the department. Except for IVF-conceived pregnancies, in all spontaneous pregnancies, more or less severe IUGR resulted in other signs of placental insufficiency, such as oligohydramnios and foetal distress. Finally, all patients delivered via caesarean section because of foetal prophylactic or vital indications. Maternal complications did not occur during pregnancy, the peripartum period, or the puerperial period.

With respect to neonatal outcomes and further infant neurodevelopment, our four pregnant TS patients had seven newborns, four girls and three boys. Prenatal and preimplantation karyotyping revealed normal 46XX or 46XY karyotypes in five patients and two karyotypes with 45,X. Severe congenital cardiovascular or other malformations were not detected preor postnatally, and the further neurodevelopmental status of the offspring was generally good, except for one infant. Specific neonatal and neurodevelopmental complications occurred only after the second pregnancy in Patient 3, when an extremely premature newborn was delivered by caesarean section. This, however, is more likely related to extreme prematurity than to TS. A 6-year-old girl suffers from somatomental retardation, is treated for hypothyroidism, suffers from ophthalmological disorders, and has short stature. At the age of 14, the child born from the first pregnancy of Patient 3 has short stature and strabism, possibly related to TS.

## DISCUSSION

Primary or premature ovarian insufficiency is a leading feature of TS. However, spontaneous pregnancy can also occur. Spontaneous menarche and mosaicism are strong predictive factors of spontaneous conception.(^3^,^14^,^15^,^18^) Whereas IVF pregnancies of TS patients and their outcomes are well documented, spontaneous pregnancies and their relationship with obstetric complications are rarely reported in the literature. In her review of the topic, Marqui identified only nine papers until 2018 reporting pregnancies of TS patients (^16^), five of which were case reports of single cases and one of which was a report of two cases. Cohorts of 53 to 480 TS patents were examined in four papers. (^2^,^14^,^19^,^20^) Calanchini et al. reported 37 spontaneous pregnancies of 18 TS patients (^3^), Ramage et al. reported 44 deliveries (^7^), and Cauldwell et al. analysed 62 spontaneous pregnancies in TS patients (^15^). In most studies, however, obstetrically significant parameters that could guide future obstetric care, such as placental insufficiency, IUGR, foetal distress and subsequent caesarean sections, are not reported. A higher frequency of small for gestational age (SGA) newborns is usually mentioned in the literature, but whether it is a result of placental dysfunction (and is therefore IUGR) or just a genetically determined but obstetrically not dangerous lower body weight is not assessed. In addition to presenting data concerning the presence of placental insufficiency, to the best of our knowledge, this is the first report in which the karyotypes of both the mothers and offspring and shortand long-term neonatal outcomes are reported at the same time, indicating that even with a normal foetal karyotype and without severe concomitant maternal diseases, TS mothers can have severely insufficiently functioning placentas, endangering their foetuses.

The rate of spontaneous conception among our TS patients (4/75; 5.3%) was similar to the rate reported in the largest cohort of a French population by Bernard et al. (27/480; 5.6%), but the rates are similar to those reported in other studies, ranging from 1.3% to 5.6%. (^2^) Our data revealed that the three patients who could conceive spontaneously experienced spontaneous menarche and subsequent irregular cycles. Two of them had TS with mosaicism, and one had a classic karyotype with 45,X monosomy.

According to previous studies, in TS pregnancies, an increased prevalence of miscarriages can be found in cases of spontaneous conception compared with the general population. This may be due to the increased rate of foetal chromosomal aberrations, but autoimmune disorders or hypoestrogenic conditions can also adversely influence obstetric outcomes. (^2^,^3^,^7^) In our cases, the known number of spontaneous miscarriages (two out of nine spontaneous pregnancies, 22.2%) was similar to the 20 to 25% rate of clinical spontaneous abortion reported in the general population, and it was somewhat lower than the rate reported in the abovementioned French cohort (30.8%). (^2^) However, not all early miscarriages are realized by these patients, as they might be less attentive to the possibility of pregnancy due to their disease. Additionally, the low number of cases does not allow us to draw firm conclusions.

Congenital cardiac anomalies can be found in nearly 50% of patients diagnosed with TS. Depending on the severity of the malformation, life-threatening complications can occur during pregnancy, e.g., aortic dissection. (^6^,^15^) Nonetheless, no severe cardiovascular complications were reported among our pregnant TS patients, although three of the four patients were followed up due to mild cardiovascular disorders.

Intrauterine growth restriction, not only SGA, was present in every spontaneous pregnancy of our TS patients. Placental insufficiency was indicated by oligohydramnios, foetal distress or both, reported as the leading indications for caesarean section. A strong correlation has been verified between gestational hypertension, ischaemic placental disease, foetal distress and premature delivery, and gestational hypertension is also more common in TS patients. (^7^,^15^) However, other factors likely played a key role in the IUGR observed in our TS pregnancies because, except for the essential hypertension of one patient (Patient 4), no other hypertensive disorder was present among our patients. It is more probable that the genetic disease itself is the source of placental insufficiency: both aneuploidy itself (^21^) and the loss of specific genes (^22^,^23^) have been suggested to cause growth restriction in TS. Additionally, our data indicate that maternal TS-related factors may be sufficient for the abnormal functioning of the placenta, as IUGR was found in both normal foetuses and TS-type foetuses. Interestingly, the only normal-weight foetus was the one conceived from a donated oocyte, which could have been produced merely by chance, but one can also hypothesize some kind of immunologic/immunotolerance background. Nevertheless, a histologically evident cause could not be identified when the cord and placenta were analysed in Patient 1.

In the case of Patient 1, a maternal ovarian biopsy was taken during the caesarean section for histologic and FISH examinations, which revealed mosaicism in the stroma cells, in line with what was previously diagnosed from peripheral lymphocytes. Additionally, histology revealed an abnormal ovarian stromal structure with few primordial follicles; however, this structure was sufficient for spontaneous ovulation and conception. Ovarian mosaicism provides an explanation for the residual reproductive potential of some patients with complete TS; interestingly, the ovarian karyotype may differ from the karyotypes of other parts of the body. (^24^) In addition to accelerated but incomplete follicular atresia (^25^), this cryptic ovarian mosaicism may be responsible for the spontaneous pregnancies of TS patients. Knowledge of ovarian and oocyte mosaicism can guide fertility preservation strategies, such as the possibility of retrieving and cryopreserving oocytes or even ovarian tissue for future use.(^26^)

We observed that the karyotypes of the foetuses were normal in the two mosaic TS patients with spontaneous pregnancies (Patients 1 and 4) and in the IVF-OD pregnancies (Patient 2). Nevertheless, in Patient 3 (45,X), who also conceived spontaneously, both foetuses were diagnosed with TS with karyotype 45,X from amniotic fluid samples. In the French cohort of Bernard et al., the karyotypes of 11 of 17 newborns from spontaneous TS pregnancies were determined, and two TS karyotypes were identified. (^2^) These data underscore the importance of both maternal and prenatal foetal karyotyping in the prediction of outcomes. Our data, however, also showed that long-term outcomes can be favourable in most cases despite the presence of maternal or foetal TS or the presence of IUGR. Moreover, with respect to the long-term neurodevelopmental well-being of the infants, the circumstances of delivery, the degree of prematurity and the birthweight were at least as important as the presence of the genetic disease. The only case with severe long-term complications was the one with an extremely low birthweight newborn of 300 g.

Approximately 1 to 5% of TS patients may have a spontaneous pregnancy, irrespective of whether the classic karyotype or mosaicism (e.g., 45,X/46,XX) of the disease is present. In these spontaneous pregnancies, SGA foetuses are common, but this seems to be caused by placental insufficiency leading to IUGR rather than just a decreased growth potential caused by the genetic disease. Placental insufficiency and IUGR may occur even if the foetus has a normal karyotype and if no maternal concomitant disease is present before conception or during pregnancy. Although the multidisciplinary medical team of TS patients must include clinicians, cardiologists and endocrinologists, even if no apparent complications are evident in these fields, obstetricians should be especially alert for foetal compromise in spontaneous TS patients’ pregnancies. If extreme prematurity and peripartum complications can be avoided, the long-term outcome of infants is good.
